# Factors related to health-related quality of life among Chinese psychiatrists: occupational stress and psychological capital

**DOI:** 10.1186/s12913-015-0677-7

**Published:** 2015-01-22

**Authors:** Chuan Liu, Lie Wang, Qun Zhao

**Affiliations:** Department of Social Medicine, School of public health, China Medical University, No.92 North second road, Heping District, Shenyang, 110001 China

**Keywords:** Psychiatrists, Health-related quality of life, Occupational stress, Psychological capital

## Abstract

**Background:**

Psychiatry has been considered as one of the most stressful medical specialities, and psychiatrists are likely to experience impaired health-related quality of life (HRQOL). However, few studies are available in regard to related factors of HRQOL among psychiatrists in China. This study aims to evaluate the condition of HRQOL of psychiatrists and explore its predictive factors, especially the effects of occupational stress and psychological capital.

**Methods:**

A cross-sectional, multicenter survey was conducted among psychiatrists from different regions of Liaoning province, China, during August 2013-April 2014. Self-administrated questionnaires including the 36-item Short-Form Health Survey (SF-36), the Chinese version Psychological Capital Questionnaire, effort-reward-imbalance (ERI) scale and participants’ basic characteristics were distributed to 500 psychiatrists from 10 psychiatric hospitals of 8 major cities in Liaoning province. Overall, 373 psychiatrists became our final research objects. Hierarchical multiple regression analysis (HMR) was performed to explore the predictors of psychiatrists’ HRQOL.

**Results:**

The mean (SD) scores of PCS and MCS among psychiatrists were 79.78 (16.55) and 71.50 (19.24) respectively. The mean (SD) of ERR were 0.777 (0.493), and 89 (23.9%) had ERR scores above 1 (ERR > 1). Hierarchical multiple regression analysis showed that, psychiatrists’ basic characteristics that significant correlated with PCS and MCS were educational level, turnover intention, and exercise; age, weekly working hours were associated with MCS; psychiatrists’ experienced occupational stress (both ERR and overcommitment), and PsyCap were significant predictors for PCS and MCS.

**Conclusions:**

Chinese psychiatrists experienced relatively good physical QOL but impaired mental QOL, and they experienced high level of occupational stress. For the sake of psychiatrists’ HRQOL, the reduction of occupational stress should be implemented. The enhancement of PsyCap could be a new intervention strategy and should be paid attention to in improving HRQOL of psychiatrists. Proportionate occupational reward (money, esteem, career opportunities) to their high work demands, psychological counseling, and stress management courses should be provided to psychiatrists to improve their QOL. PsyCap, as a personal coping resource open to change, should be managed and developed among psychiatrists.

## Background

Psychiatry has been considered as one of the most stressful medical specialities as it is frequently dealing with chronically ill, often incurable patients with mental illness [1]. Most of the studies reported in China have been generally focused on physicians, while there are relative fewer studies specifically concerning the physical and mental quality of life (QOL) of psychiatrists. Psychiatrists reported less often “good” or “rather good” self-perceived health [2]. Violence and the fear of violence [3], dealing with difficult and hostile relatives of patients, managing suicidal or homicidal patients [4], poorly defined roles of consultants, responsibility without authority [5] and high efforts into work with fewer rewards from psychiatric patients than other patients make psychiatry a more stressful profession than other physicians and surgeons. Previous researches reported that psychiatrists suffered from burnout, depression and mental disorder more commonly than other physicians [2,6,7]. They are more vulnerable to vicarious trauma, compassion fatigue and have higher level of suicidal tendencies [8-10]. These issues can have major impact not only on psychiatrists’ service delivery and quality of care, but also their own health-related quality of life (HRQOL).

According to Siegrist’s effort-reward-imbalance (ERI) at work model, occupational stress has been described as being caused by a negative effort-reward imbalance reflecting a disproportion between effort and reward (money, esteem, career opportunities) plus overcommitment (an excessive work-related commitment) at work [11]. Mental health professions like psychiatrists faced additional strain by the very nature of their professions in dealing with troubled persons often over extended periods of time [12]. Overwork, relationship problems with other staff, performance-related stress, organization problems, inadequate resources, lack of planning, and threats to self-esteem, as main stressors in psychiatrists [13], are very likely to lead to occupational stress among psychiatrists. Occupational stress could induce reduced well being and increased illness susceptibility [14].

Psychological capital (PsyCap) is a kind of positive psychological state of an individual in the process of growth and development and is a higher-order core construct drawn from positive organizational behavior [15]. Luthans and his colleagues identified that the positive psychological constructs meeting the inclusion criteria so far included hope, resilience, optimism, and self-efficacy, and represented PsyCap when combined [16,17]. These four state-like psychological resources can all be measured, developed and managed [18,19]. PsyCap has been reported as a positive resource for improving employee’s well-being over time [20], and for combating employee’s occupational stress and turnover [15], burnout and depressive symptoms among Chinese doctors [21,22].

There have been studies conducted to investigate the health and its related factors of psychiatrists in different countries [2,7,23,24]. However, to our knowledge, few studies have been conducted to explore the related factors of HRQOL among psychiatrists in China so far. So, this study aims to evaluate the condition of HRQOL of Chinese psychiatrists and explore its predictive factors, especially the effects of occupational stress and PsyCap. This study may provide evidence and theoretical basis for developing strategy to improve QOL of psychiatrists.

## Methods

### Study design and sample

A cross-sectional, multicenter survey was conducted in Liaoning province, located in northeastern China, from August 2013 to April 2014. The survey was cooperated by 10 main psychiatric hospitals from 8 major cities of Liaoning province. A total of 500 Self-administered questionnaires were sent to the aforementioned 10 psychiatric hospitals, and distributed to psychiatrists with the help of the administrative staff of each hospital. All the participants were totally voluntary and anonymously, and they were orally informed about the contents and the aim of the questionnaire. After reading the informed consent, Self-administered questionnaires were directly distributed to participants and they completed the questionnaires with no interference. Effective responses were obtained from 373 psychiatrists (effective response rate: 74.6%) after excluding questionnaires that had not been correctly completed. The sample represents about 37.0% of the psychiatrists in Liaoning province, the total number of psychiatrists in which is merely about 1000. The study protocol and informed consent form were in accordance with the standard of the Committee on Human Experimentation of China Medical University and received approval.

### Measurements

#### Psychiatrists’ basic characteristics

Demographic characteristics regarding gender, age in years, marital status, and educational level were included. Information of work situation including monthly income, job title, weekly working hours, and turnover intention were obtained. “Marital status” was categorized as “single”, “married/cohabitation” and “divorced/others”. “Educational level” was categorized as “Junior college or lower”, “Undergraduate” and “Graduate or higher”. “Monthly income (RMB)” was categorized as “<3000”, “3000-4999”, and “≥5000”. “Weekly working hours” was categorized as “≤40 hours” and “>40 hours”. Job title was categorized as “Senior (chief physician/professor, associate chief physician/associate professor)”, “Intermediate (attending doctor)”, “Junior (resident doctor)”, and “None”.

#### Health behavior and life events

Respondents were asked to answer how many times they exercise per week (divided into three categories: no, once a week, two times a week or more), and whether they drink or smoke or not (Yes/No). As for life events, respondents were asked a simple dichotomous question: “Have you experienced any life events (i.e. death, illness, or laid-off of kinsfolk, entering a higher school or getting married of children) during the past year? (Yes/No)”.

#### Health-related quality of life (HRQOL)

The 36-item Short-Form Health Survey (SF-36) was used to assess HRQOL. The SF-36 measures eight different concepts of health: physical functioning (PF), role limitations due to physical problems (RP), bodily pain (BP), general health (GH), vitality (VT), social functioning (SF), role limitations due to emotional problems (RE), and mental health (MH), which were selected from the Medical Outcomes Study inventory [25]. These eight dimensions can be aggregated into physical component summary (PCS) and mental component summary (MCS). A score was calculated for each dimension and was transformed to obtain a value ranging from 0 to 100, with higher scores indicating better health [26]. The Mandarin version of SF-36 is a valid and reliable tool for assessing HRQL [27]. The Cronbach’s alpha for PCS and MCS in this study were 0.862 and 0.868, respectively.

#### Occupational stress

Occupational stress was measured according to Siegrist’s effort-reward-imbalance (ERI) at work model in which chronic work-related stress is identified as non-reciprocity or imbalance between high efforts spent and low rewards received [28]. It is a 23-item ERI scale that consists of three scales termed extrinsic effort (6 items), reward (11 items), and overcommitment (6 items), and the Chinese version of the scale has been widely used in China with good reliability [22,29-31]. The measuring procedure of effort and reward includes two steps: firstly, participants have to express their attitudes (agree or disagree) to the work condition described by the item; then, they are asked to choose the extent to which they feel distressed (from “not distressed” to “very distressed”). Overcommitment was assessed with 6-item form on a 4-point rating scale, and participants expressed to what extent they agree or disagree with the given statements. For the ERI scale, occupational stress can be expressed by effort/reward ratio (ERR) and overcommitment independently. The ERR score was calculated based on the following equation: ERR = 11 × effort/6 × reward. When ERR becomes >1, a high amount of effort not met with adequate reward is indicated. High score on overcommitment indicates the tendency to spend an inadequate amount of effort not met by externally defined reward. In the present study, the Cronbach’s alpha for extrinsic effort, reward, and overcommitment scales was 0.914, 0.898 and 0.702 respectively.

#### Psychological capital

PsyCap was measured with the Chinese version of the 24-item Psychological Capital Questionnaire (PCQ) developed by Luthans et al. [32]. Each of the four dimensions of PsyCap (self-efficacy, hope, resilience and optimism) is measured by six items scored on a 6-point Likert scale (1 represents strongly disagree and 6 represents strongly agree). An average score for the total scale was calculated to get a composite PsyCap construct, with a higher value indicated higher level of experienced PsyCap. The Chinese version of PCQ has been used in various Chinese studies with satisfactory reliability [21,22]. In this study, Cronbach’s alpha for self-efficacy, hope, resilience and optimism was 0.880, 0.906, 0.786, and 0.657 respectively. For the total scale, the Cronbach’s alpha was 0.935.

#### Statistical analysis

Differences of physical and mental QOL in categorical variables were examined by two sample t-test and one-way ANOVA. Pearson’s correlation was performed to test the correlation among continuous variables. With QOL scores (PCS and MCS) as dependent variables, Hierarchical Multiple Regression analysis was performed to test the incremental variance by any given set of independent variables. In the present study, characteristics of psychiatrists were added to the regression model in the first step, ERR and overcommitment were added in the second step, and PsyCap was added in the third step. The relative importance of the variables retained in the final multiple regression models contributed to the explained variance of the QOL, which was represented as the standardized β [33]. Adjusted R^2^-value was used to assess the fit of the model. A p value <0.05 (two-sided) was considered as statistically significant. All data were analyzed with SPSS v13.0 statistical program for windows. All study variables were standardized before analysis and we used tolerance and variance inflation factor to check for multicollinearity.

## Results

### Participant characteristics

Effective responses were obtained from 373 psychiatrists. The basic characteristics of participants are provided in Table 1. The mean ± SD age of participants was 37.89 (9.64) years, ranging from 24 to 86 years. Most psychiatrists (N = 226, 60.6%) were women. As for marital status, the majority (75.9%) of participants were married/cohabitation, 18.5% were single, and 5.6% were divorced/others. Most respondents (N = 265, 71.0%) have completed undergraduate college and only 8.6% had graduate degree or above. With respect to job title, 66 (17.7%) had senior title, 152 (40.8%) had intermediate title, and 108 (29.0%) had junior title. A relative small number of participants were drinkers (N = 59, 15.8%) or smokers (N = 55, 14.7%). More baseline characteristics of participants were presented in Table 1.

**Table 1:** **Characteristics of psychiatrists, means and standard deviations (SDs) of PCS and MCS**

**Variable**	**N (%)**	**PCS (Mean ± SD)**	**MCS (Mean ± SD)**
**Total**	373(100)	79.78 ± 16.55	71.50 ± 19.24
**Gender**			
Male	147(39.4)	79.31 ± 17.33	70.95 ± 20.16
Female	226(60.6)	80.08 ± 16.05	71.88 ± 18.66
**Age**			
≤30	103(27.6)	79.81 ± 16.55	71.53 ± 18.51
31-40	128(34.3)	80.13 ± 16.66	70.84 ± 18.23
41-50	111(29.8)	79.49 ± 16.92	70.92 ± 20.91
>50	31(8.3)	79.30 ± 19.36	76.23 ± 19.72
**Marital status**			
Single	69(18.5)	80.69 ± 16.22	73.10 ± 17.46
Married/cohabitation	283(75.9)	79.78 ± 16.66	71.17 ± 19.45
Divorced/Others	21(5.6)	76.79 ± 16.49	70.72 ± 22.53
**Educational level**			
Junior college or lower	76(20.4)	76.50 ± 18.60	71.11 ± 20.33
Undergraduate	265(71.0)	79.96 ± 16.08	70.70 ± 19.05
Graduate or higher	32(8.6)	86.11 ± 13.33*	79.11 ± 16.96*
**Monthly income (RMB)**			
<3000	125(33.5)	78.72 ± 17.78	70.76 ± 20.44
3000-4999	217(58.2)	80.31 ± 15.86	71.61 ± 18.49
≥5000	31(8.3)	80.38 ± 16.48	73.77 ± 19.87
**Job title**			
Senior	66(17.7)	83.26 ± 15.01	75.06 ± 17.06
Intermediate	152(40.8)	78.15 ± 17.23	70.07 ± 20.03
Junior	108(29.0)	79.35 ± 15.48	70.41 ± 19.08
None	47(12.6)	81.16 ± 18.33	73.68 ± 19.65
**Weekly working hours**			
≤40	225(60.3)	81.61 ± 15.96**	74.58 ± 17.68**
>40	148(39.7)	76.99 ± 17.08	66.83 ± 20.60
**Turnover intention**			
Never	194(52.0)	84.15 ± 13.84***	77.17 ± 17.40***
Sometimes	149(39.9)	77.67 ± 16.36	67.87 ± 18.73
Frequently	30(8.0)	61.95 ± 19.80	52.87 ± 17.38
**Exercises**			
No	154(41.3)	76.62 ± 18.60**	66.85 ± 21.03**
Once a week	92(24.7)	81.48 ± 14.14	74.13 ± 18.06
two times a week or more	127(34.0)	82.38 ± 15.09	75.25 ± 16.58
**Drinking**			
No	314(84.2)	80.16 ± 16.05	71.77 ± 19.17
Yes	59(15.8)	77.77 ± 19.02	70.10 ± 19.72
**Smoking**			
No	318(85.3)	80.29 ± 15.95	72.07 ± 18.91
Yes	55(14.7)	76.80 ± 19.57	68.23 ± 20.94
**Life events**			
No	230(61.7)	82.05 ± 15.81**	73.93 ± 19.18**
Yes	143(38.3)	76.12 ± 17.11	67.61 ± 18.76

*p < 0.05, **p < 0.01, ***p < 0.001.

PCS: physical component summary; MCS: mental component summary.

### Description of psychiatrists’ HRQOL

In the present study, the mean ± SD scores of PCS and MCS among psychiatrists were 79.78 ± 16.55 and 71.50 ± 19.24 respectively (Table 1). MCS scores were significantly lower than that of PCS. Mean ± SD scores of PCS and MCS based on the characteristics of psychiatrists were also showed in Table 1. Gender differences of PCS and MCS were not spotted. Psychiatrists with graduate or higher degree reported significantly higher PCS and MCS than those with lower educational level. Those psychiatrists who worked more than 40 hours a week or had experienced life events in the last year had significant lower PCS and MCS. Moreover, psychiatrists that never had turnover intention had significant higher PCS and MCS scores than those who sometimes or frequently did. And those regularly did exercise showed significantly better PCS and MCS than those never did. Differences of PCS and MCS between drinkers and non-drinkers and between smokers and non-smokers were not found.

### Correlations among variables and predictors of psychiatrists’ HRQOL

The mean and logarithmic mean (SD) of ERR were 0.777 (0.493) and −0.188 (0.262) (Table 2) and 89 (23.9%) had ERR scores above 1 (ERR > 1). In the Pearson correlation analysis (Table 2), both ERR and overcommitment were significantly negatively correlated with PCS and MCS, while PsyCap was significantly positively correlated with PCS and MCS.

**Table 2:** **Means, standard deviations, and correlation among study variables**

	**Mean**	**SD**	**1**	**2**	**3**	**4**
1. ERR	−0.188	0.262				
2. Overcommitment	15.201	3.436	0.586**			
3. PsyCap	4.348	0.723	−0.414**	−0.223**		
4. PCS	79.780	16.548	−0.408**	−0.332**	0.489**	
5. MCS	71.504	19.243	−0.508**	−0.413**	0.556***	0.756**

**p < 0.01, ***p<0.001.

ERR: effort/reward ratio; PsyCap: psychological capital; PCS: physical component summary; MCS: mental component summary.

The mean of ERR is logarithmic (mean ± SD of ERR was 0.777 ± 0.493).

The results of hierarchical multiple regression models of PCS and MCS were presented in Table 3 and Table 4. In the final regression model, 33.5% of the variance of PCS, and 44.9% of MCS were explained. Psychiatrists’ basic characteristics, occupational stress, and PsyCap explained 19.7%, 8.7%, and 8.6% of the variance of PCS, respectively; and explained 22.9%, 13.5%, and 11.4% of the variance of MCS, respectively. In the regression model, psychiatrists’ basic characteristics that significant correlated with PCS and MCS were educational level, turnover intention, and exercise; age, weekly working hours were associated with MCS; psychiatrists’ experienced occupational stress (both ERR and overcommitment), and PsyCap were significant predictors both for PCS and MCS.

**Table 3:** **Hierarchical multiple regression predicting the PCS scores**

**Variable**		**PCS**		
		**Step1(** **β)**	**Step2(β** **)**	**Step3(** **β)**
**Basic characteristics**				
Age		0.099	0.062	0.036
Gender	(Man vs. Women)	0.024	0.031	0.041
Marital status				
	(Single vs. Married/cohabitation)	0.001	−0.038	−0.033
	(Divorced/others vs. Married/cohabitation)	−0.052	−0.057	−0.046
Educational level				
	(Undergraduate vs. junior college or lower)	0.137*	0.134*	0.077
	(Graduate or higher vs. junior college or lower)	0.158**	0.136*	0.093
Income		−0.062	−0.034	−0.024
Job title				
	(Senior vs. junior)	0.083	0.093	0.078
	(Intermediate vs. junior)	−0.017	−0.002	0.009
Weekly working hours		−0.076	0.012	0.019
Turnover intention				
	(Never vs. frequently)	0.585***	0.437***	0.324***
	(Sometimes vs. frequently)	0.421***	0.370***	0.319***
Exercise (times per week)		0.124*	0.095*	0.078
Drinking	(Yes vs. no)	−0.024	−0.018	−0.034
Smoking	(Yes vs. no)	−0.025	0.013	0.019
Life events	(Yes vs. no)	−0.085	−0.084	−0.092*
**Occupational stress**				
ERR			−0.256***	−0.140*
Overcommitment			−0.128*	−0.142**
**PsyCap**				0.337***
Adjusted R^2^		0.197***	0.247***	0.335***
ΔR^2^		0.197	0.087	0.086

*p < 0.05, **p < 0.01, ***p < 0.001.

ERR: effort/reward ratio; PsyCap: psychological capital; PCS: physical component summary.

ERR is logarithmic transformed in the model.

**Table 4:** **Hierarchical multiple regression predicting the MCS scores**

**Variable**		**MCS**		
		**Step1(β** **)**	**Step2(β** **)**	**Step3(** **β)**
**Basic characteristics**				
Age		0.168*	0.121	0.092
Gender	(Man vs. Women)	0.007	0.016	0.028
Marital status				
	(Single vs. Married/cohabitation)	0.043	−0.004	0.002
	(Divorced/others vs. Married/cohabitation)	−0.026	−0.031	−0.019
Educational level				
	(Undergraduate vs. junior college or lower)	0.075	0.070	0.004
	(Graduate or higher vs. junior college or lower)	0.133*	0.105	0.056
Income		−0.084	−0.050	−0.039
Job title				
	(Senior vs. junior)	0.073	0.086	0.069
	(Intermediate vs. junior)	−0.010	0.011	0.023
Weekly working hours		−0.163**	−0.052	−0.045
Turnover intention				
	(Never vs. frequently)	0.506***	0.322***	0.192*
	(Sometimes vs. frequently)	0.295**	0.232**	0.173*
Exercise (times per week)		0.176***	0.140**	0.121**
Drinking	(Yes vs. no)	0.009	0.018	−0.002
Smoking	(Yes vs. no)	−0.058	−0.011	−0.005
Life events	(Yes vs. no)	−0.058	−0.056	−0.066
**Occupational stress**				
ERR			−0.331***	−0.198***
Overcommitment			−0.144**	−0.161**
**PsyCap**				0.387***
Adjusted R^2^		0.195***	0.332***	0.449***
ΔR^2^		0.229	0.135	0.114

*p < 0.05, **p < 0.01, ***p < 0.001.

ERR: effort/reward ratio; PsyCap: psychological capital; MCS: physical component summary.

ERR is logarithmic transformed in the model.

## Discussion

The results of our study indicate that psychiatrists’ physical QOL was slightly better than Chinese general population, while mental QOL was relatively poorer [34]. Chinese psychiatrists were experiencing high level of occupational stress. Occupational stress and PsyCap were significant predictors of HRQOL of psychiatrists.

We found that Chinese psychiatrists are experiencing relatively good physical QOL, but impaired mental QOL, which is consistent with previous studies [7,23]. As mentioned before, high level of psychological distress, serious job burnout, and depression are commonly seen among psychiatrists. And, in one study dedicated on psychiatric nurses, Pompili et al. provided some insight on the role of burnout and psychodynamic mechanisms at work in causing hopelessness and suicide risk among those more engaged in stressful activities with psychiatric patients [35]. Considering the similar working environment of psychiatrists, this may also explain the reason for psychiatrists’ poor mental health. So it is reasonable that impaired mental QOL was found among psychiatrists. Although some studies suggested that doctors do not adequately take care of their own physical health [36,37], compared with general population, doctors have a low level of mortality rate [38], and they were more likely to practice disease prevention [39]. Doctors, with their specialty of diagnosing and treating disease, may have better health maintenance behavior and are more sensitive to the change of their physical condition than general population, which may be also an explanation for their relatively better physical QOL.

In this study, psychiatrists’ basic characteristics explained 19.7% variance of PCS and 22.9% variance of MCS. The regression analysis showed that turnover intention was the strongest predictor of psychiatrists’ physical and mental QOL among all the basic characteristics. Psychiatrists who sometimes or frequently have turnover intentions reported worse physical and mental QOL than those who never have. In previous studies, it has been reported that turnover intention was negatively correlated with job satisfaction, organizational commitment, job stress and quality of work life among hospital employees [40,41]. Furthermore, employees who have turnover intention are likely to have burnout and emotional exhaustion [42,43]. These may induce impairment to physical and mental health.

Educational level was another important predictive factor of QOL of psychiatrists, and psychiatrists with higher education background tended to have better physical and mental QOL. Due to the characteristics of medical education in China, Chinese doctors are having varying degree of education. Compared with those with lower education degree, doctors with higher education background have higher prestige among colleagues, more career opportunities, better remuneration under the same or maybe less workload, which may be the reason why they are relatively healthier.

Age was also significantly positively related to psychiatrists’ mental QOL. Tyssen R reported in a review that perceived overwork and emotional pressure were predictive factors of young doctors’ mental health [44]. By contrast, for older doctors, it is generally believed that with age comes higher level of occupational skills and expertise. In China’s healthcare environment, age equals to doctors’ qualifications and record of service, older doctors have higher position in the minds of patients and colleagues, and higher sense of achievement from work. So, they are facing less pressure from work, which would contribute to their relatively good mental state.

Doctor's work is characterized by heavy work during the day, accompanied with frequent night shifts. They often deal with patients with extended periods of time and have little control over their work schedules, which is even worse in China due to its enormous population. In this study it is found that weekly working hour was significant related to psychiatrists’ QOL. In the regression model, psychiatrists with more weekly working hours showed poorer mental health. This is consistent with previous studies which reported that extended hours of work was positively related to ill-health [45,46]. Therefore, hospitals should establish standardized diagnosis and treatment system, and formulate scientific and reasonable work schedule so as to improve the work efficiency and reduce work load of psychiatrists.

Psychiatrists’ level of physical and mental QOL rises with the increase of times of exercises per week. Due to the nature of their work, psychiatrists often live sedentary lifestyle, which is associated with deleterious health outcomes [47]. There is a substantial literature indicating that regular exercise could lead improvement in physical and mental QOL [48]. So, Psychiatrists should adopt regular exercise in their daily lives. Life events and health behaviors like drinking and smoking were not significant related to psychiatrists’ QOL in our regression analysis.

In the present study, 23.9% psychiatrists had ERR scores above 1 (ERR > 1), with a mean (SD) of 0.777 (0.493), which is higher than the 0.66 (0.34) of Chinese healthcare providers (physicians and nurses) [49], that of Chinese workers with coronary heart disease [29], and that of Chinese physicians [30], and is higher than the 19.3% found in German psychiatrists [50]. These implied that Chinese psychiatrists were experiencing high level of occupational stress. And we also confirmed that occupational stress was a strong predictor of both physical and mental QOL of psychiatrists, and explained 8.7% and 13.5% of the variance of PCS and MCS, respectively. The more occupational stress (high effort, low reward, and overcommitment) psychiatrists experienced during work, the worse their physical and mental QOL were.

Occupational stress has been recognized globally as a major health hazard for employees. Our finding was in consistent with general view we have from medicine and psychiatry on occupational stress and its consequences. For example, previous studies reported that occupational stress (ERI model) could induce worsening physical and mental conditions, including burnout [51], cardiovascular diseases [52], psychiatric disorders [53], and psychosomatic symptoms [54]. Among Chinese physicians, the association between effort-reward imbalance and impaired health functioning was also confirmed [30].

In order to improve the HRQOL of psychiatrists, as well as the quality of healthcare service, it is crucial to take relative measures to prevent and reduce the occupational stress that psychiatrists experience during work. For example, adequate salary, appropriate work load, respect and support, job security, career opportunities like promotion, and so on, are aspects that organizations should take into account to increase the occupational reward of psychiatrists, so as to alleviate their emotional distress from work. Psychiatrists and doctors in general often fail to receive adequate treatment of mental health problems [55,56]; they are reluctant to seek advice formally from their colleagues [57]. Therefore, psychological counseling should been provided to psychiatrists so that their emotional stress as well as other mental health problems can be treated promptly and properly. Moreover, stress management courses and occupational interventions [58], extra support during life events, complaints and when a patient dies by suicide [7], are also effective ways.

PsyCap was also a significant predictor of psychiatrists’ HRQOL, explaining 8.6% and 11.4% of the variance of PCS and MCS, respectively. Psychiatrists who experience higher PsyCap tend to have better physical and mental QOL. As mentioned before, PsyCap, composed of four positive psychological constructs (hope, resilience, optimism, and self-efficacy), has been found to be a positive resource for improving employee’s well-being, and for combating employee’s occupational stress and turnover, burnout and depression. As regards health care workers, high PsyCap increases constructive emotions, reduces destructive emotions and eventually increase well-being [59], and PsyCap also predict better work performance, better work well-being (job satisfaction, physical/psychological symptoms), and better social well-being (work-life balance, quality of life) [60], which also support our results. In light of these findings, the development of PsyCap and its four components could be a new intervention strategy to improve HRQOL of psychiatrists. PsyCap, as a personal coping resource, should be paid attention to in improving the physical and mental QOL of psychiatrists.

Several limitations of the present study must be mentioned. Firstly, we employed a cross-sectional design, which did not allow us to make any casual statements about the relationships between the variables investigated. Another potential limitation is that we used a single source of self-reported data in our research. Common method variance may artificially inflate the relationships between variables. Multiple data collecting methods and longitudinal approach should be used in further research. Thirdly, we initially focused our attention on psychiatrists from main specialized hospitals for psychosis, while those worked in psychiatric department of various levels of general hospitals were not included. What’s more, we did not collect the characteristics of those psychiatrists who refused to participate in the survey and the reason why they refused to. These relatively “inactive” psychiatrists may be those more affected by worse quality of life. So our data may not comprehensively reflect the condition of HRQOL of Chinese psychiatrists. Finally, all the participants were from only one province. In consideration of the vast territory of China and district economic imbalance, it may be difficult to generalize the results to all psychiatrists across the country.

## Conclusions

Chinese psychiatrists experienced relatively good physical QOL but impaired mental QOL. The level of occupational stress was high among psychiatrists. Occupational stress and PsyCap were significant predictors for physical and mental QOL of psychiatrists. The reduction of occupational stress should be implemented for the sake of psychiatrists’ HRQOL. The enhancement of PsyCap could be a new intervention strategy and should be paid attention to in improving HRQOL of psychiatrists. Proportionate occupational reward (money, esteem, career opportunities) to their high work demands, psychological counseling, and stress management courses should be provided to psychiatrists to improve their QOL. PsyCap, as a personal coping resource open to change, should be managed and developed among psychiatrists.

## Abbreviations

QOL: Quality of life
HRQOL: Health related quality of life
SF-36: The 36-item short-form health survey
PCS: Physical component summary
MCS: Mental component summary
PsyCap: Psychological capital
